# Clinical characteristics of *Clostridium difficile*-associated diarrhea among patients in a tertiary care center in China

**DOI:** 10.12669/pjms.323.9400

**Published:** 2016

**Authors:** Yongqiang Li, Yi Huang, Yuyuan Li, Yuqiang Nie

**Affiliations:** 1 Yongqiang Li, Department of Gastroenterology, Guangzhou Digestive Diseases Center, Guangzhou First People’s Hospital, Guangzhou Medical University, Guangdong, China; 2 Yi Huang, Department of Gastroenterology, Guangzhou Digestive Diseases Center, Guangzhou First People’s Hospital, Guangzhou Medical University, Guangdong, China; 3 Yuyuan Li, Department of Gastroenterology, Guangzhou Digestive Diseases Center, Guangzhou First People’s Hospital, Guangzhou Medical University, Guangdong, China; 4 Yuqiang Nie, Department of Gastroenterology, Guangzhou Digestive Diseases Center, Guangzhou First People’s Hospital, Guangzhou Medical University, Guangdong, China

**Keywords:** Antibiotic-associated diarrhea, *Clostridium difficile*, Incidence, Risk factors

## Abstract

**Objective:**

This study investigated the incidence, risk factors, and clinical characteristics of *Clostridium difficile*-associated diarrhea (CDAD) in Chinese patients.

**Methods:**

Fecal specimens of patients with antibiotic-associated diarrhea (AAD) were collected to test *C. difficile* toxin A and B using enzyme-linked fluorescent assay to identify CDAD. By adopting a nested case-control design, the matched people (ratio 1:3) without AAD were included as controls.

**Results:**

Out of 56,172 inpatients, 39,882 (71.0%) used antibiotics, 470 suffered from AAD, and 93 were diagnosed with CDAD. The incidence of nosocomial CDAD was 166 per 100,000. The proportion of CDAD in AAD was 19.8%. CDAD patients presented with more severe clinical manifestations and exhibited more concurrent illness. Logistic regression analysis showed the risk factors of CDAD: advanced age, nasogastric tube-feeding, high APACHE II scores, high level of serum C-reaction protein, low level of serum albumin, severe underlining disease or comorbidity, and number of antibiotic intake. Twenty-nine patients (31.2%) were cured with vancomycin, 54 (58.1%) were cured after dual therapy of vancomycin plus metronidazole, 7 (7.5%) died of underlying diseases aggravated with CDAD, and 3 (3.2%) were transferred to other hospitals for personal reasons.

**Conclusion:**

The incidence of nosocomial CDAD in China was high. Some risk factors could predispose CDAD.

## INTRODUCTION

Clostridium difficile (*C. difficile*)-associated diarrhea (CDAD) is a common cause of infectious diarrhea in hospitals, which usually occurs as a complication of antibiotic therapy. CDAD is becoming a growing worldwide health threat with an incidence of approximately 100 per million and a mortality of 1%–2.5% in western countries.^1^ Thus, controlling the spread of this infection is urgently needed.

The clinical spectrum of cases presented with CDAD can be extensive, which range from asymptomatic carriage to mild self-limiting diarrhea and more severe pseudo-membranous colitis. Accurate diagnosis of CDAD is crucial in managing individual patients. Over the past two decades, rapid diagnostic methods through testing *C. difficile* toxin (CDT) were developed to detect *C. difficile* infection. Currently, commercial assays to detect both CDT A and B are available with reasonable sensitivity and specificity. Obtaining information of the *C. difficile* infection worldwide is crucial, following the dramatically increasing rate of CDAD and the recent emergence of the new highly virulent strains of *C. difficile* in Canada, USA, and Europe.^2^-^5^ However, the data in China are not well documented. In present study, we studied the incidence, risk factors, and clinical characteristics of CDAD in Chinese patients.

## METHODS

The study included hospitalized patients admitted to this hospital between April 1, 2008 and March 31, 2010. The patients who exhibited diarrhea after being administered antibiotics for at least 3 days were selected according to the diagnostic criteria issued by Health Ministry of China,^6^ which was adapted from the guideline of American College of Gastroenterology.^7^ These patients suffered from antibiotic-associated diarrhea (AAD) and were included in this study. The diagnosis of AAD was based on the clinical manifestations, i.e., abdominal cramps, profuse diarrhea (bowel movements > three times/day with mucoid, greenish, foul-smelling, and watery stools or pseudo-membrane), low-grade fever, and leukocytosis, which presented several days after initiating antibiotic therapy.

Other gastrointestinal diseases, e.g., bacterial and amebic dysentery, typhoid fever, food poisoning, inflammatory bowel disease, irritable bowel syndrome, lactose intolerance, and colorectal cancer must be ruled out before diagnosis. Stool examinations and colonoscopy were needed when the diagnosis was suspected. Fecal specimens were collected from each patient for the *C. difficile* toxin assay. The patient was diagnosed with CDAD when the result of the assay was positive. A case was considered complicated if one or more of the following was observed: megacolon, perforation, colectomy, shock requiring vasopressor therapy, or death within 30 days after diagnosis. The cases of AAD without CDAD were set as the control. With a ratio of 1:3 for each CDAD cases, 279 matched patients (age ±5 years and same gender) from the same department who received antibiotics for at least three days, but had no diarrhea during the same period of time were selected as another control group. The study was approved by the Ethics Committee of the hospital. Written consent was obtained from each participant.

### Interview and physical examination

The study was prospectively designed. Face-to-face interview and physical examination were carried out for each subject by specially trained post-graduate students of Guangzhou Medical University and supervised by experienced investigators. Standard questionnaires including demographic data, current medication use, medical history, and health-relevant behaviors, i.e., alcohol consumption, smoking habits, and dietary habits, were recorded. The patients were followed up for an interval of three days during their stay at the hospital, and their clinical data from patient charts and hospital computer databases were collected for analysis.

### Detection of C. difficile

Fresh fecal specimens were collected and sent to the laboratory within 2 hours. The tests for *C. difficile* toxin A and B were performed immediately by utilizing enzyme-linked fluorescent assay kits (bioMérieux, France). The tests were carried out according to the instruction of the kits (bioMérieux mini-VIDAS standard). The stool specimens were diluted and centrifuged. The supernatant fluid was placed in the holes of testing kits with CDAD reagent strips inside. The results were measured by bioMérieux equipment. The positive threshold value was set at 0.13. A value < 0.13 was considered negative for CDAD, which suggested AAD. Meanwhile, a value of ≥ 0.13 was considered positive, which may imply CDAD.

### Variables of observation

The outcomes of interest included the following variables: 1. general conditions: temperature, respiratory rate, heart rate, blood pressure, and consciousness; 2. clinical manifestation: severity and frequency of abdominal pain, frequency of bowel movements, appearance of stools, and abdominal tenderness; 3. laboratory parameters: routine analysis of blood, urine, and stools, liver and kidney function tests, C-reaction protein, and blood gas analysis; 4. administration of medicines: antibiotics, corticosteroid, and immunosuppressive agents; 5. pass history and underlining diseases; and 6. therapeutic intervention: nasogastric tube-feeding, urethral catheterization, and tracheal cannula. The Acute Physiology and Chronic Health Evaluation II (APACHE II) score system was employed to further evaluate the severity of the disease.^8^

### Statistical analysis

The data were analyzed with SPSS 17.0 (Chicago, IL, USA). The continuous data were expressed as mean±SD and examined by Student’s t-test. Categorical variables were presented as a percentage and examined by χ^2^ tests and Fisher’s tests. Statistical significance was set at *P* < 0.05 (two-tailed). Exposure ratio comparison and multivariate regression analyses were performed to evaluate the risk factors.

## RESULTS

This general hospital with 1,400 patient beds is located at downtown of Guangzhou, a large city in southern China with population of 10,045,800. Only patients with advanced diseases are usually admitted to the hospital because of the shortage of medical resources. Between April 1,2008 and March 31, 2010, 56,172 patients were admitted, of which 39,882 (71.0%) used antibiotics for at least three days. A total of 470 were diagnosed with AAD, among which 93 had CDAD (males, 61; females, 32; aged 68±16 years old) and 377 had AAD without CDAD (males, 211; females, 166; aged 67±19 years old). The incidence of nosocomial CDAD was 166 per million in this hospital. The proportions of AAD and CDAD among inpatients receiving antibiotics were 11.8% and 0.23%, respectively. The proportion of CDAD in AAD was 19.8%. The hospital does not serve a well-defined population. Thus, we were unable to calculate the population-based incidence.

### Clinical characteristics of CDAD

Among total 93 patients with CDAD, 70 (75.3%) were elderly (>60 years old). The main underlying diseases of the patients included respiratory failure with 23 cases (24.7%), heart failure with 14 cases (15.1%), cerebrovascular accidence with 12 cases (12.9%), gastrointestinal diseases with 16 cases (17.2%), leukemia with 11 cases (11.8%), post-abdominal operation with 8 cases (8.6%), and others with 9 cases (9.7%). Seventy-one of the patients (76.3%) exhibited concurrent diseases, such as infection, chronic obstructive lung disease, diabetes mellitus, hypertension, heart insufficiency, renal insufficiency, and malnutrition. Forty-two (45.2%) of the patients had more than one concurrent disease. Forty patients (43%) had nasogastric feeding. The average APACHE scores of CDAD patients upon diagnosis were 16.6±4.3. Diarrhea occurred 4–39 days after antibiotic intake. The frequencies of bowel movement were 4–10 per day. Liquid stools were observed in 39 cases (41.9%); mushy stools in 27 cases (29.0%); mucous stools in 16 cases (17.2%), and bloody purulent stools in 11 cases (11.8%), among which pseudo-membranes were found in three cases (3.2%).

Compared with patients with AAD, the patients with CDAD presented significantly longer durations of hospitalization and antibiotic intake, higher serum level of C-reactive protein, lower level of serum albumin, and higher scores of APACHE II and comorbidity (mostly in the lung, heart, liver, and kidney) (Table-I) (*P* < 0.05). The species and numbers of antibiotics were not significantly different between the two groups (*P* > 0.05) except glucopeptide (mainly vancomycin) and nitroimidazole (mainly metronidazole), which protected AAD patients from progressing to CDAD (*P* < 0.05) (Table-I). The multivariate regression logistic analysis demonstrated that C-reactive protein, duration of hospitalization, APACHE II scores, and comorbidity were risk factors (Table-II).

**Table-I:** Clinical characteristics of CDAD patients compared with AAD.

*Variables*	*CDAD*	*AAD*	*P value*
N	93	377	
Age (yrs)	68±16	67±19	NS*
Duration of hospitalization	18.5±10.4	15.2±13.4	< 0.05
Duration of antibiotics	10.9±7.1	8.8±6.3	< 0.05
Nasogastric feeding (n)	40 (45%)	142 (37.6%)	NS
Comorbidity (n)	73 (78.5%)	150 (39.8%)	< 0.05
WBC (×10^12^/l)	9.1±3.4	8.6±4.2	NS
C-reactive protein (mg/l)	34.4±35.2	23.4±25.8	< 0.05
Creatinine (umol/l)	151.9±113.4	132.7±25.6	NS
Albumin (g/l)	29.3±5.4	32.0±1.8	< 0.05
APACHE II scores	16.6±4.4	11.5±5.3	< 0.05
*Intake of antibiotics*				
Third generation cephalosporin	50 (53.8%)	175 (46.4%)	NS
Second generation cephalosporin	20 (21.5%)	80 (21.2%)	NS
Quinolinones	39 (41.9%)	144 (38.2%)	NS
Macrolide	20 (21.5%)	77 (20.4%)	NS
Aminoglycoside	25 (26.9%)	88 (23.3%)	NS
Carbopenems	15 (16.1%)	58 (15.4%)	NS
Glucopeptide#	2(2.2%)	36 (9.5%)	< 0.05
Nitroimidazole+	10(10.8%)	81 (21.5%)	< 0.05
*Quantity of antibiotics*				
Mono	20 (21.5%)	86 (22.8%)	NS
Dual	58 (62.4%)	220 (58.4%)	NS
Triple	15 (16.1%)	71 (18.8%)	NS

CDAD: *C difficile* associated diarrhea;

AAD: Antibiotic-associated diarrhea.

* NS: not significant;

# including vancomycin;

+ including metronidazole, ornidazole.

**Table-II:** Multivariate regression logistic analysis for CDAD in AAD patients.

*Variables*	*RR*	*95% CI*	*P value*
Duration of hospitalization	2.40	(1.59, 3.63)	< 0.01
Duration of antibiotics	0.91	(0.64, 1.29)	0.603
C-reactive protein	2.73	(1.58, 4.7)	< 0.01
Albumin	1.34	(0.81, 2.21)	0.250
Comorbidity	5.52	(3.23, 9.44.)	< 0.01
APACHE II scores	6.53	(3.65, 11.68)	< 0.01
Usage of glucopeptide or nitroimidazole	3.00	(1.59, 5.79)	< 0.01

*RR: relative risk; CI: confidence interval.

Compared with those of the controls, the variables significantly predisposing CDAD were advanced age, high WBC, nasogastric feeding, high C-reactive protein, high serum albumin level, comorbidity, and APACHE II scores (*P* < 0.05) (Table-III). The multivariate regression logistic analysis showed that nasogastric feeding, C-reactive protein, APACHE II, serum albumin level, and comorbidity were risk factors for CDAD (Table-IV).

**Table-III:** Clinical characteristics of CDAD patients compared with controls.

*Variables*	*CDAD*	*Controls*	*P value*
N		93	279	
Nasogastric feeding (n)	40 (45%)	42 (15.1%)	<0.05
Age (yrs)	68±16	59.7±22.6	<0.05
Comorbidity (n)	73 (78.5%)	117 (41.9%)	<0.05
WBC (×10^12^/l)	9.1±3.4	8.37±2.8	<0.05
C-reactive protein (mg/l)	34.4±35.2	13.8±15.5	<0.05
Creatinine (umol/l)	151.9±113.4	94.1±75.1	<0.05
Albumin (g/l)	29.3±5.4	34.5±5.7	<0.05
PACHE II scores	16.6±4.3	9.7±4.4	<0.05

**Table-IV:** Multivariate regression logistic analysis for the risk of CDAD in inpatients.

*Variables*	*β*	*SE*	*Wald*	*RR (95%CI)*	*P value*
Nasogastric feeding	1.449	0.268	29.20	4.26 (2.52,7.20)	< 0.05
Comorbidity	1.620	0.281	33.47	5.05 (2.92,8.75)	< 0.05
APACHE II	2.44	0.408	35.91	11.52 (5.18,25.63)	< 0.05
C-reactive protein	0.938	0.257	13.36	2.55 (1.55,4.22)	< 0.05
Albumin	0.801	0.247	10.52	2.23 (1.37,3.6)	< 0.05
Numbers of antibiotics	0.294	0.286	1.056	1.34 (0.766,2.352)	0.304
Third generation cephalosporin	0.779	0.243	10.257	2.18(1.35,3.51)	< 0.05
Quinolinones	0.679	0.250	7.357	1.97(1.21,3.22)	< 0.05
Glucopeptide	-1.596	0.743	4.615	0.203(0.05,0.87)	< 0.05
Nitroimidazole	-1.112	0.361	9.475	0.33(0.16,0.67)	< 0.05

### The impact of antibiotics selection on CDAD

Compared with those of the controls, the administration of the third generation cephalosporin, quinolones significantly increased the incidence of CDAD after administering the third generation cephalosporin. However, glucopeptide and nitroimidazole significantly decreased the incidence of CDAD (Table-V).

**Table-V:** Antibiotics intake in CDAD and control groups.

*Antibiotics*	*CDAD*	*Control*	*χ^2^*	*P*
*Used antibiotics*				
Third generation cephalosporin (n)	50 (53.8%)	96 (34.4%)	10.96	<0.05
Second generation cephalosporin (n)	20 (21.5%)	76 (27.2%)	1.20	NS
Quinolinones (n)	39 (41.9%)	74 (26.5%)	7.83	<0.05
Macrolide (n)	20 (21.5%)	80 (28.7%)	1.82	NS
Aminoglycoside (n)	25 (26.9%)	60 (21.5%)	1.14	NS
Carbopenems	15 (16.1%)	24 (8.6%)	4.21	<0.05
Glucopeptide (n)	2(2.2%)	27 (9.7%)	5.50	<0.05
Nitroimidazole (n)	10(10.8%)	74 (26.5%)	9.92	<0.05
*Number of antibiotics*				
Mono	20 (21.5%)	75 (26.8%)	1.06	NS
Dual	58 (62.4%)	176 (63.1%)	0.02	NS
Triple	15 (16.1%)	28 (10.0%)	2.53	NS

*NS: >0.05

### Prognosis of patients with CDAD

Discontinuation of antibiotics was possible in 49 patients. Ninety-three patients with CDAD were given 0.25–0.5 g Vancomycin four times a day. After the 7 day treatment, 29 patients (31.2%) were cured. The poor responders were administered 200–400 mg Metronidazole four times a day. The remaining 54 patients (58.1%) were cured with dual therapy, among which 38 (40.9%) responded within the next 7 days and 11 responded within 27 days. Seven patients (7.5%) died of underlying diseases aggravated with CDAD. Three patients (3.2%) were referred to other hospitals for personal reasons.

## DISCUSSION

There are several laboratory techniques to identify *C. difficile* infection. However, the best standard method has not been clearly established. *C. difficile* cytotoxin neutralization and toxigenic culture are usually considered as the primary reference tests. However, these tests are time consuming and require equipment and expertise. Stool culture alone is not recommended because not all *C. difficile* strains are toxigenic. According to the new guidelines published by AJG in 2013,^9^ Nucleic acid amplification tests (NAAT) such as polymerase chain reaction (PCR) for *C. difficile* toxin genes appear to be sensitive and specific and may be used as a standard diagnostic test for CDI. *C. difficile* glutamate dehydrogenase (GDH) test is a sensitive test with low specificity. This test is applied as a screen test to eliminate most samples (that test negative) for further testing.^10^ GDH may be used in association with toxin A and B enzyme immunoassay (EIA) testing. EIA for toxin A/B used to be the most widely used diagnostic test because it is fast, inexpensive, and has high specificity. Although its sensitivity is lower than that of NAAT, EIA can identify the toxigenic strains, which are of clinical importance.^11^ In this study, we focused on the pathogenic strains of *C. difficile*. Thus, EIA for *C. difficile* toxin A/B was chosen. Moreover, this testing approach was suggested by UK consensus at that time.^12^

CDAD became prevalent in the 1960s and 1970s with the introduction of broad-spectrum antibiotics to the clinical practice. The incidence of *C. difficile* infection increased rapidly. During the middle and late 1990s, the reported incidence of *C. difficile* infection in acute care hospitals in the United States remained stable at 30 to 40 cases per million in population. In 2001, this rate increased to almost 50, with subsequent increases to 92.8 in 2011.^2^ The outbreaks of *C. difficile* infection in 2003 with increased severity of illness were reported in Quebec, Canada with an incidence of 156.3 per million, and 102.0 to 866.5 among patients aged 65 years old or more. The outbreaks were caused by a new virulent strain (called PCR ribotype 027).^13^ Later, many parts of the United States reported similar outbreaks caused by the same strain;^14^,^15^ the outbreaks spread to Europe afterward.^16^ Reported mortality rates from CDAD in the United States increased from 5.7 per million in 1999 to 23.7 per million in 2004. The rate increased to 1%–2.5% in 2008 because of the emergence of a highly virulent strain of *C. difficile*.^1^ The incidence and mortality rate of *C. difficile* infection in other places, particularly in developing countries, have not been documented. The present study reveals a high incidence of nosocomial CDAD (166 per million) at the hospital in Guangzhou, China. Although we were unable to calculate population-based incidence and determine the strain of *C. difficile*, these results should warrant more attention in the investigation of CDAD.

Identifying patients who are at high risk for CDAD early in the course of their infection may help improve outcomes, although the predictors are not well known. Compared with AAD patients, CDAD patients usually present more severe clinical manifestations and have more concurrent illnesses.^17^ Laboratory markers, such as leukocytosis, increased creatinine levels, and decreased albumin levels, were reported to correlate with the development and poor outcome for patients with CDAD.^18^ Disrupting normal intestinal flora by antibiotics is a well-known mechanism for CDAD. Thus, antibiotic intake is the most important risk factor. Other drugs, such as immunosuppressive agents and proton pump inhibitors, are also significant risk factors for CDAD precipitation. Although the antibiotics most frequently implicated in the predisposition to *C. difficile* infection are fluoroquinolones, clindamycin, cephalosporins, and penicillins, virtually all antibiotics (including metronidazole and vancomycin) can predispose the patient to *C. difficile* infection. Risk is higher when the patients are on multiple antibiotics and undergo longer courses of therapy.^19^ In this study, no difference was observed in most antibiotic intakes between AAD and CDAD groups. The administration of glucopeptide (mainly vancomycin) and nitroimidazole were even negatively related to CDAD (as protective factors). In non-drug risk factors, nasogastric feeding, comorbidity, elevated serum C-reactive protein, and creatinine levels, decreased serum albumin, and increased APACHE II scores were all risk factors of developing CDAD in comparison with that of the controls. However, compared with that of AAD, only comorbidity, elevated serum C-reactive protein, duration of hospitalization, and increased APACHE II scores were the risk factors of CDAD. This group of patients shared most non-drug risk factors of those reported in developed countries. This outcome implied that the host responses to the infection of Chinese and their western counterparts were identical.

The usual treatment for CDAD was to stop administration of antibiotics meant for other purposes and to immediately start treatment with metronidazole or vancomycin. Patients who remain on antibiotics while undergoing CDAD treatment are likely to experience metronidazole treatment failure.^20^ In this study, administering glucopeptide (mainly vancomycin) and nitroimidazole was a protective factor, and most patients were successfully cured with these two kinds of drugs probably because patients were infected with the strain of *C. difficile* sensitive to the drugs. The resistant strain of *C. difficile* seemed uncommon in China.

In conclusion, to our knowledge, the present work is a rare prospective study that investigates the incidence and clinical characteristics of CDAD in mainland of China.^21^-^25^ We determined a high incidence of *C. difficile* infection in China. The major limitations of this study were the comparatively small sample size and the testing method. This work still provides vital information, which may provide basis for future research focusing on the prevention of CDAD.
